# Micro-RNAs in regenerating lungs: an integrative systems biology analysis of murine influenza pneumonia

**DOI:** 10.1186/1471-2164-15-587

**Published:** 2014-07-11

**Authors:** Kai Sen Tan, Hyungwon Choi, Xiaoou Jiang, Lu Yin, Ju Ee Seet, Volker Patzel, Bevin P Engelward, Vincent T Chow

**Affiliations:** Department of Microbiology, Yong Loo Lin School of Medicine, National University Health System, National University of Singapore, 5 Science Drive 2, Kent Ridge, 117545 Singapore; Infectious Diseases Interdisciplinary Research Group, Singapore-Massachusetts Institute of Technology Alliance in Research and Technology, Kent Ridge, 138602 Singapore; Saw Swee Hock School of Public Health, National University of Singapore, Kent Ridge, 117597 Singapore; Department of Pathology, National University Hospital, Kent Ridge, 119074 Singapore; Department of Medicine, University of Cambridge, Cambridge, CB2 0SP UK; Department of Biological Engineering, Massachusetts Institute of Technology, Cambridge, MA 02139 USA

**Keywords:** Lung repair, Pulmonary regeneration, Influenza pneumonia, miRNAs, miRNome, Transcriptome

## Abstract

**Background:**

Tissue regeneration in the lungs is gaining increasing interest as a potential influenza management strategy. In this study, we explored the role of microRNAs, short non-coding RNAs involved in post-transcriptional regulation, during pulmonary regeneration after influenza infection.

**Results:**

We profiled miRNA and mRNA expression levels following lung injury and tissue regeneration using a murine influenza pneumonia model. BALB/c mice were infected with a sub-lethal dose of influenza A/PR/8(H1N1) virus, and their lungs were harvested at 7 and 15 days post-infection to evaluate the expression of ~300 miRNAs along with ~36,000 genes using microarrays. A global network was constructed between differentially expressed miRNAs and their potential target genes with particular focus on the pulmonary repair and regeneration processes to elucidate the regulatory role of miRNAs in the lung repair pathways. The miRNA arrays revealed a global down-regulation of miRNAs. TargetScan analyses also revealed specific miRNAs highly involved in targeting relevant gene functions in repair such as miR-290 and miR-505 at 7 dpi; and let-7, miR-21 and miR-30 at 15 dpi.

**Conclusion:**

The significantly differentially regulated miRNAs are implicated in the activation or suppression of cellular proliferation and stem cell maintenance, which are required during the repair of the damaged lungs. These findings provide opportunities in the development of novel repair strategies in influenza-induced pulmonary injury.

**Electronic supplementary material:**

The online version of this article (doi:10.1186/1471-2164-15-587) contains supplementary material, which is available to authorized users.

## Background

### Vulnerability of lungs to influenza virus infection

Our lungs are constantly in contact with the external environment and exposed to airborne debris, pathogens and chemicals, which can inflict damage acutely or chronically [1]. Pathogens such as influenza virus can cause acute lung injury (ALI) through acute viral pneumonia [2]. ALI induced by influenza may also lead to complications that severely impair pulmonary functions, and not only affect an individual’s quality of life, but can also be life-threatening if left untreated [2–5]. Furthermore, with the emergence of pandemic H1N1-2009, avian H5N1 and H7N9 influenza strains, and seasonal H1N1 and H3N2 strains, which cause varying severity of ALI, there is a need for novel and timely intervention strategies for post-influenza lung repair to mitigate permanent tissue damage and its sequelae. Among available options, promoting pulmonary tissue regeneration represents an effective approach against influenza-induced lung injury [6, 7].

### Regenerative medicine as a novel influenza management strategy

Influenza infection of the lungs constitutes an important source of severe inflammatory damage to the lung architecture via respiratory burst of the innate immune response that cannot be generalized to other types of lung injury [5, 8]. The injury can give rise to complications and/or chronic damage if not managed properly, particularly in susceptible groups such as the elderly and allergic individuals with diminished repair functions to cope with these infections [3, 4]. Being a common respiratory pathogen, influenza has therefore been extensively investigated to elucidate its infection kinetics and pathogenicity by clinical studies and animal models [9, 10]. Hence, with better understanding of its kinetics, novel strategies can be formulated based on the phases of the influenza infection process. The repair stage of influenza pneumonia is gaining increasing interest given that complications can be prevented if the damaged lung architecture is restored appropriately. Studies on the recovery period of pulmonary influenza infection have indicated changes in proliferative and differentiation traits of lung epithelial cells [11, 12], suggesting potential regulation of these changes to facilitate the repair of damaged lungs.

### MicroRNAs as potential agents in post-influenza lung repair strategies

MicroRNAs (miRNAs) are short RNA nucleotides involved in post-transcriptional regulation, and are of major interest in view of their ease of manipulation and delivery into host cells due to their relatively small sizes [13–15]. In addition, they regulate various disease processes as well as normal physiologic functions, including embryonic lung development [13–20]. The recent success of miRNA-based drug discovery [21] further fuelled our interest to screen for miRNAs that mediate repair of influenza-afflicted lungs. We hypothesized that miRNAs play major roles in priming pulmonary tissues for repair and regeneration following influenza pneumonia, similar to how miRNAs are intimately involved in embryonic lung development [16, 17].

This study aimed to investigate miRNAs that are differentially regulated during the repair phases of influenza using an established murine model of influenza H1N1 pneumonia, drawing parallels to seasonal H1N1 infection that may culminate in pulmonary complications in susceptible individuals. Another objective was to attain complete systems-level analysis to elucidate potential miRNAs that are crucial for lung repair *in vivo* [22]. These findings can improve our understanding of influenza-induced lung repair responses, and also identify miRNA candidates for further validation as critical players in pulmonary regeneration.

## Results

### Infected murine lungs exhibit similar degrees of damage, while miRNA and mRNA microarray expression profiles are largely homogenous at specific time-points

The lungs on 7 days post-infection (dpi) were scored using a modified damage scoring system [23] to ascertain that the mice suffered similar extents of damage, while lungs on 15 dpi were scored based on the area of recovery. Lungs that displayed similar degrees of injury within each individual time-point at 7 and 15 dpi were subjected to miRNA and mRNA microarrays (Table 1). Pearson correlation was computed between all samples to compare the homogeneity of miRNA and mRNA expression within each animal group, which showed that miRNA and mRNA expression profiles were generally highly correlated. For subsequent analyses, only the probe-set intensity data that were relatively homogenous between replicates of the specific infected groups at 7 and 15 dpi were included. Thus, consistent miRNA profiles from 3 mice at 7 dpi and 4 mice at 15 dpi, together with consistent mRNA profiles from 4 mice each at 7 and 15 dpi were analyzed (Additional file 1: Figure S1).

**Table 1:** **Damage scoring of infected murine lungs**

***Days & mice***	***% lung affected***	***Alveolar septae***	***Hemorrhage***	***Fibrin***	***Infiltrate***	***Score***
***7 dpi***						
Infected 1	30	3	1	1	3	3.9
Infected 2	40	3	1	1	3	5.2
Infected 3	30	3	1	1	3	3.9
Infected 4	50	3	1	1	3	6.5
***15 dpi***			***% lung affected***			
Infected 1			60			
Infected 2			70			
Infected 3			60			
Infected 4			40			

Note: Histopathologic scoring to determine the homogeneity of lung damage at 7 and 15 dpi. The formula to derive the scores at 7 dpi is:% infected × [alveolar hemorrhage + 2(alveolar inflitrates) + 3(fibrin) + alveolar septal congestion].

### Cell types, degree of cell proliferation and repair in the lungs vastly differ between 7 and 15 dpi

**Figure 1:**
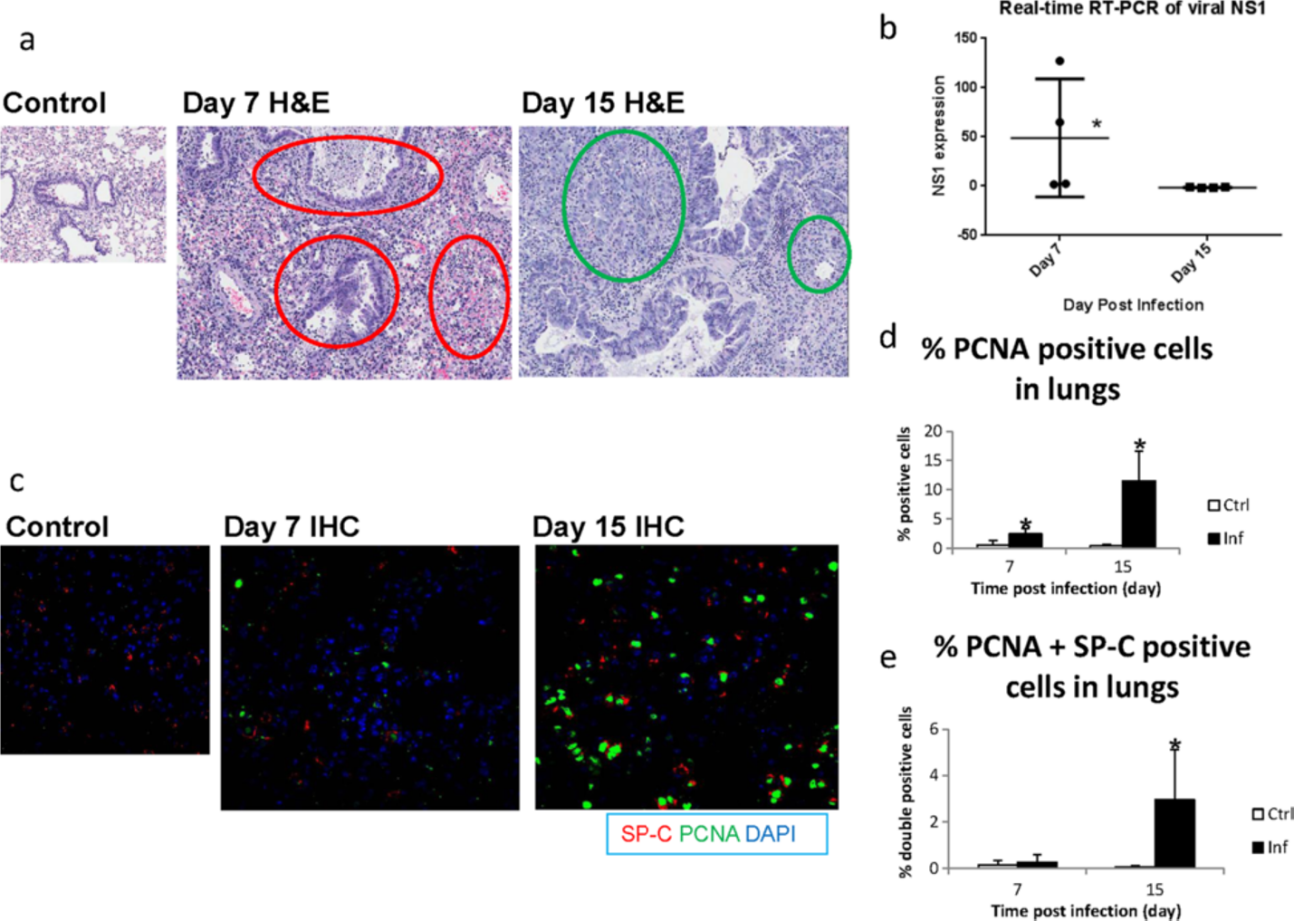
**Histopathologic and IHC analyses of murine lungs.** **(a)** The histopathologic images of infected lungs (H&E) portray considerably greater extent of epithelial thickening and infiltration into the lungs at 7 dpi than 15 dpi (red circles). At 7 dpi, minimal repair processes were observed other than wound healing (fibrin). In comparison, at 15 dpi, new epithelial and connective tissue filled the alveolar spaces (green circles). **(b)** Expression of viral NS1 at 7 dpi indicated presence of virus, whereas complete viral clearance was evident at 15 dpi. **(c)** Dividing cells (PCNA-positive) and AT2 pneumocytes (SP-C-positive) were stained by IHC to assess lung regeneration at both time-points. **(d)** Higher percentages of PCNA-positive proliferating cells were noted in infected lungs at 7 dpi and especially at 15 dpi. **(e)** PCNA and SP-C double-positive dividing cells were significantly higher in infected murine lungs at 15 dpi. N = 4 per group per time-point. Asterisks denote P < 0.05 compared to uninfected control.

Compared to uninfected control, histopathologic analyses of infected lungs (Figure 1a) revealed more damaged areas, and a greater number of infiltrating polymorphs at 7 dpi, suggesting predominant innate immune responses against the virus in the lungs. This finding is congruent with the viral NS1 expression, which was still detected in lungs at 7 dpi (Figure 1b). Most areas of the lungs exhibited widening interstitia and septae, with the presence of fibrin and red blood cells. In contrast, at 15 dpi, while some features of broncho-alveolar damage remained (i.e. apoptotic debris, desquamation), viral expression was absent in the lungs. Furthermore, the most striking change was the appearance of new epithelial cells at 15 dpi. Also, the subjective increase in all types of infiltrating cells on 15 dpi is likely to account for the apparent increase in area of lung damage compared with 7 dpi.

Immunohistochemistry (IHC) analyses with proliferating cell nuclear antigen (PCNA) and surfactant protein-C (SP-C) staining were also conducted to ascertain the degree of cellular proliferation, DNA synthesis and repair in the lungs (specifically for alveolar type 2 or AT2 pneumocytes). Minimal PCNA-positive cells were present in the control mice at both days, while SP-C-positive AT2 pneumocytes were evenly distributed across the lungs (Figure 1c). Lungs of infected mice at 7 dpi displayed areas without SP-C-positive cells likely attributed to virus-induced damage. Increased PCNA-positive cells were observed in the infected lungs. However, there was only a small percentage of PCNA and SP-C double-positive cells at 7 dpi, which was not significant compared to control uninfected lungs (Figure 1d, e). On 15 dpi, multiple areas deprived of SP-C-positive cells were likely attributed to pneumocytes damaged by infection and innate immune responses. Relatively more PCNA-positive cells as well as PCNA and SP-C double-positive cells were observed in the lungs at 15 dpi, indicating enhanced cell proliferation, DNA synthesis and DNA repair of the pneumocytes.

### General down-regulation of miRNAs and up-regulation of genes at repair phases on both 7 and 15 dpi

**Figure 2:**
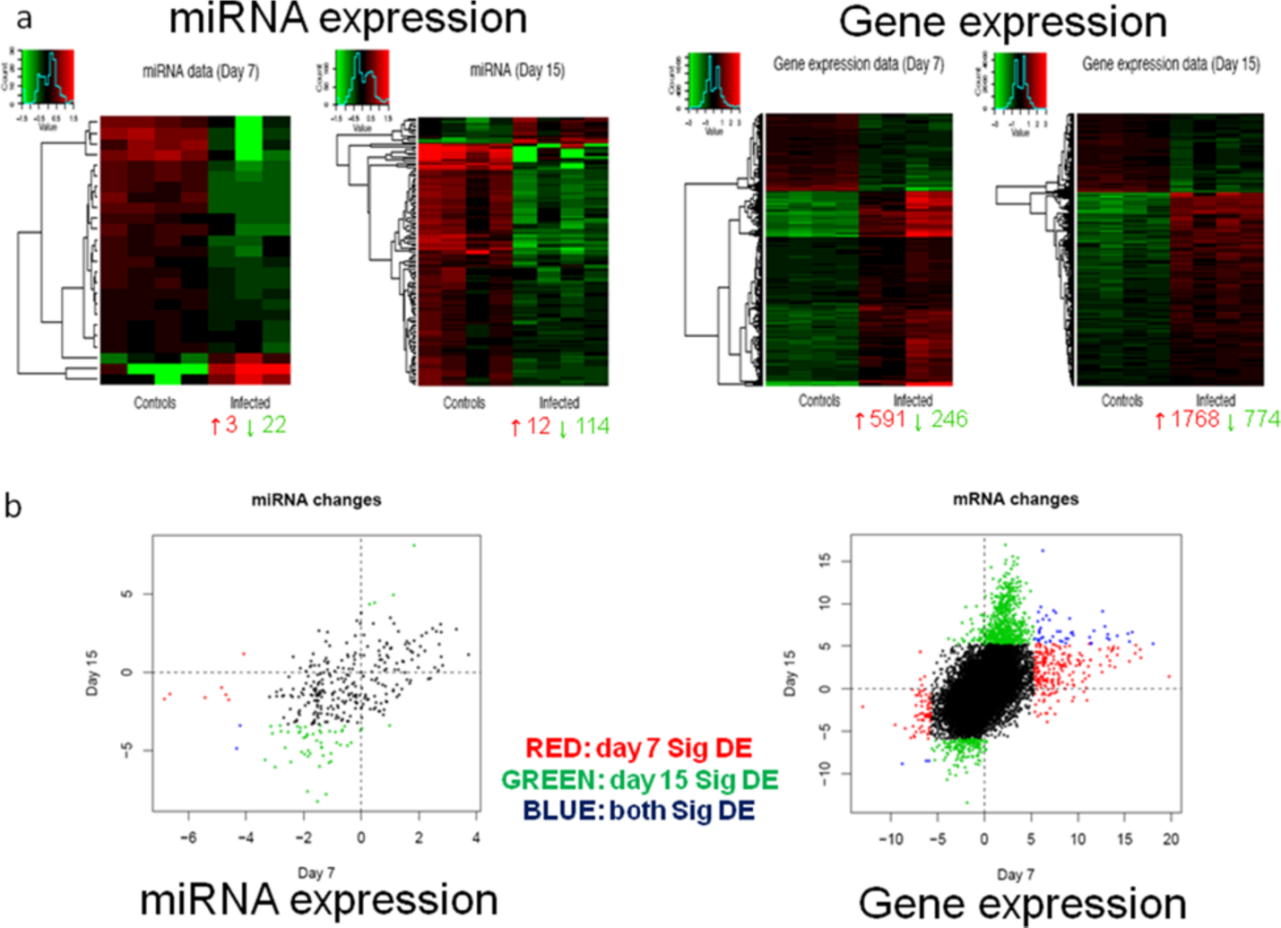
**microRNA and gene microarray expression on 7 and 15 dpi. (a)** Representative heatmaps of microarrays revealed that relatively fewer miRNAs and genes were differentially expressed at 7 dpi. However, more alterations in expression occurred at 15 dpi, when viral clearance was complete. In infected lung samples at both 7 and 15 dpi, the higher numbers of down-regulated miRNAs coincided with the increase in up-regulated genes. **(b)** Scatter plots showed negligible overlap between the differentially expressed miRNAs and genes at the two time-points, indicating that highly specific functions were operating during these two phases of influenza pneumonia. N = 4 per group per time-point.

The miRNA array data on both 7 and 15 dpi revealed a global down-regulation of miRNAs (at false discovery rate or FDR of 5%). On 7 dpi, 3 miRNAs were up-regulated and 22 miRNAs were down-regulated out of 336 miRNAs analyzed. However, on 15 dpi, 12 miRNAs were up-regulated and 114 down-regulated out of 314 miRNAs analyzed (Figure 2a). Table 2 outlines some of the top significant miRNAs on both days. This landscape suggests that miRNAs, being gene regulatory factors via translational repression, were down-regulated to yield augmented gene expression in response to H1N1 infection. Indeed, the mRNA expression patterns were skewed towards up-regulation in the infected groups, with 591 up-regulated genes and 246 down-regulated genes at 7 dpi (out of 36,991 genes analyzed); and 1,768 up-regulated genes and 744 down-regulated genes at 15 dpi (out of 35,577 genes analyzed). In comparison, infected mice revealed ~5 times more differentially expressed (DE) miRNAs (at least 1.3× fold change) and ~3 times more DE genes (at least 1.5× fold change) on 15 dpi compared to 7 dpi. This indicates more significant alterations in expression at 15 dpi, presumably due to complete viral clearance. There was almost no overlap between the DE miRNAs and genes between the two time-points, implying that the biological processes were largely distinct due to the different conditions of the infected lungs at each time-point (Figure 2b).

**Table 2:** **Significantly differentially expressed (DE) murine miRNAs at 7 and 15 dpi**

***Day 7 miRNA***	***Log***_***2***_ ***fold***	***FDR***
mmu-let-7b-3p	-1.98	0.000000
mmu-miR-486-3p	-1.61	0.000000
mmu-miR-20a-3p	-1.47	0.000028
mmu-let-7f-1-3p	-1.67	0.000293
mmu-miR-1940	1.50	0.000395
mmu-miR-191-3p	-1.34	0.000538
mmu-miR-505-3p	-1.24	0.000808
mmu-miR-144-5p	-1.06	0.002555
mmu-miR-582-3p	-1.52	0.004199
mmu-miR-335-3p	-1.97	0.007209
mmu-miR-200b-5p	-0.98	0.044629
mmu-miR-290-5p	2.10	0.049478
***Day 15 miRNA***	***Log***_***2***_ ***fold***	***FDR***
mmu-miR-21-5p	2.53	0.000000
mmu-miR-34c-3p	-2.35	0.000000
mmu-miR-34b-3p	-2.55	0.000000
mmu-miR-676-3p	-2.31	0.000000
mmu-miR-322-3p	-2.04	0.000000
mmu-miR-10b-5p	-1.24	0.000000
mmu-miR-145-5p	-1.37	0.000001
mmu-miR-450b-3p	-2.50	0.000001
mmu-miR-181a-5p	-1.36	0.000001
mmu-miR-200b-5p	-2.22	0.000001
mmu-miR-30b-3p	-1.86	0.000005
mmu-miR-181c-5p	-1.40	0.000005
mmu-miR-151-5p	-1.26	0.000008
mmu-miR-375-3p	-1.96	0.000011
mmu-miR-542-5p	-2.93	0.000011
mmu-miR-193-3p	-1.83	0.000017
mmu-miR-30e-3p	-1.44	0.000025
mmu-miR-144-5p	-1.49	0.000041
mmu-miR-3107-5p	-1.80	0.000052
mmu-miR-30c-2-3p	-1.83	0.000058
mmu-miR-1843-5p	-1.19	0.000064
mmu-miR-34c-5p	-1.33	0.000094
mmu-miR-29c-5p	-1.16	0.000106
mmu-miR-30a-3p	-1.36	0.000199
mmu-miR-10a-5p	-0.94	0.000203
mmu-miR-574-3p	-1.52	0.000217
mmu-miR-155-5p	2.19	0.000299
mmu-miR-328-3p	-1.52	0.000347
mmu-miR-194-5p	-1.07	0.000649
mmu-miR-192-5p	-1.01	0.000767

Note: The top 12 DE miRNAs on day 7, and the top 30 DE miRNAs on day 15 are listed. miRNAs with at least 1.3× fold change and P-value < 0.05 were considered significantly DE.

**Figure 3:**
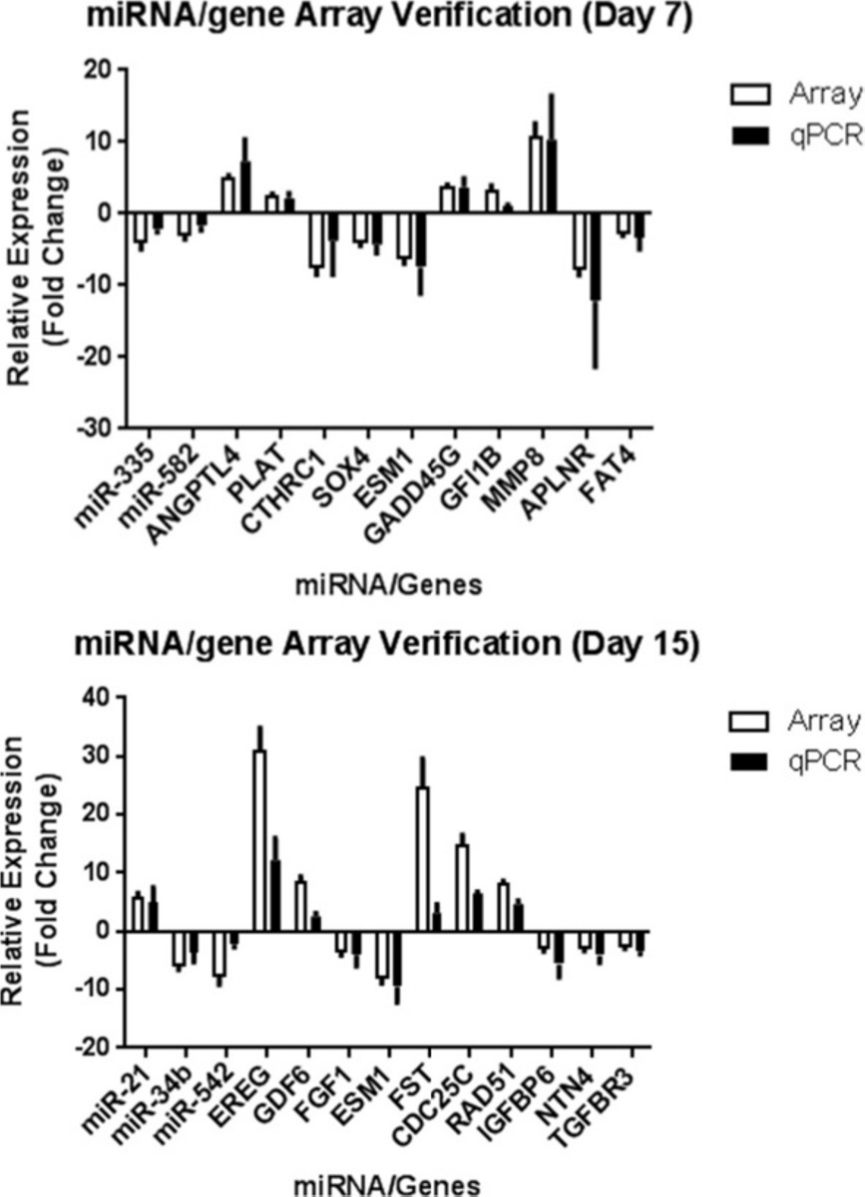
**Real-time RT-PCR verification of microarray data.** Verification of microarray data by real-time RT-PCR (qPCR) assays revealed congruent results for virtually all the 5 miRNAs and 20 genes tested for both time-points. These results confirmed the accuracy and validity of the microarrays performed. N = 4 per group per time-point.

Real-time RT-PCR data of representative significantly DE miRNAs and genes indicated good consistency with the microarray expression data (Figure 3). Thus, both miR-335 and miR-582 were down-regulated at 7 dpi; while miR-21 was up-regulated, miR-34b and miR-542 were down-regulated at 15 dpi. For gene expression at 7 dpi, genes such as *ANGPTL4* and *PLAT* were up-regulated, while genes such as *SOX4* and *CTHRC1* were down-regulated. At 15 dpi, genes such as *EREG* and *GDF6* were up-regulated, while genes such as *ESM1* and *FGF1* were down-regulated. The directional trends of changes in all the DE genes were consistent with the array data, thus validating the accuracy of both miRNA and gene microarrays.

### Overall global gene networks by Ingenuity pathway analysis (IPA)

Table 3 shows the top 5 IPA networks of the DE genes for each time-point. At 7 dpi, functions and pathways such as immune cell proliferation, inflammation, and innate viral responses were significantly enriched (P < 0.05), while repair functions were relatively sparse. The number of functions and pathways was much lower at 7 dpi likely due to viral presence and immediate innate immune responses to combat the virus, thus masking other functions. On the other hand, following viral clearance by 15 dpi, innate immune responses were likely to be largely replaced by more influenza-specific adaptive responses that are more tightly controlled and regulated. Thus, the pathway enrichment shifted from immune responses to largely cellular responses of repair and proliferation, such as DNA damage response, cellular proliferation, DNA replication and cell cycle activities (P < 0.05).

**Table 3:** **Ingenuity pathway analysis of enriched pathways during different repair phases**

***Day 7 networks***	***Score***
Hematological system development and function, Tissue morphology, Cellular development	52
Antimicrobial response, Inflammatory response, Infectious Disease	40
Cell-to-cell signaling and interaction, Cellular growth and proliferation, Hematological system development and function	37
Cardiovascular system development and function, Cell morphology, Hematological system development and function	31
Cell-to-cell signaling and interaction, Cellular function and maintenance, Hematological system development and function	31
***Day 15 networks***	***Score***
Nucleic acid metabolism, Small molecule biochemistry, Cellular development	42
Cell-to-cell signaling and interaction, Cell signaling, Nucleic acid metabolism	40
DNA replication, recombination, and repair, Cell cycle, Cellular assembly and organization	36
Cell death and survival, Cell-to cell signaling and interaction, Hematological system development and function	35
Cellular compromise, DNA replication, recombination and repair, Energy production	35

Note: IPA analysis based on gene function enrichment. Enrichment score is a modified P-value and indicates the likelihood of the Focus Genes in a network being found together due to random chance. A score of 2 denotes that there is a 1-in-100 chance that the Focus Genes are together in a network due to random chance. Therefore, scores of 2 or higher have at least 99% confidence of not being generated by random chance alone.

### Repair pathway selection and gene ontology (GO) analyses

To focus on miRNA regulatory activities in the relevant biological functions, we applied the IPA function tree to manually select genes that were annotated in repair, development and regeneration functions. Thus, 227 and 876 genes were selected for further analyses for days 7 and 15 (out of a total of 837 and 2,512), respectively. In this selection, the DE genes were mainly involved in cell death, cell survival, cell cycle, cell proliferation, DNA replication, and developmental functions.

**Figure 4:**
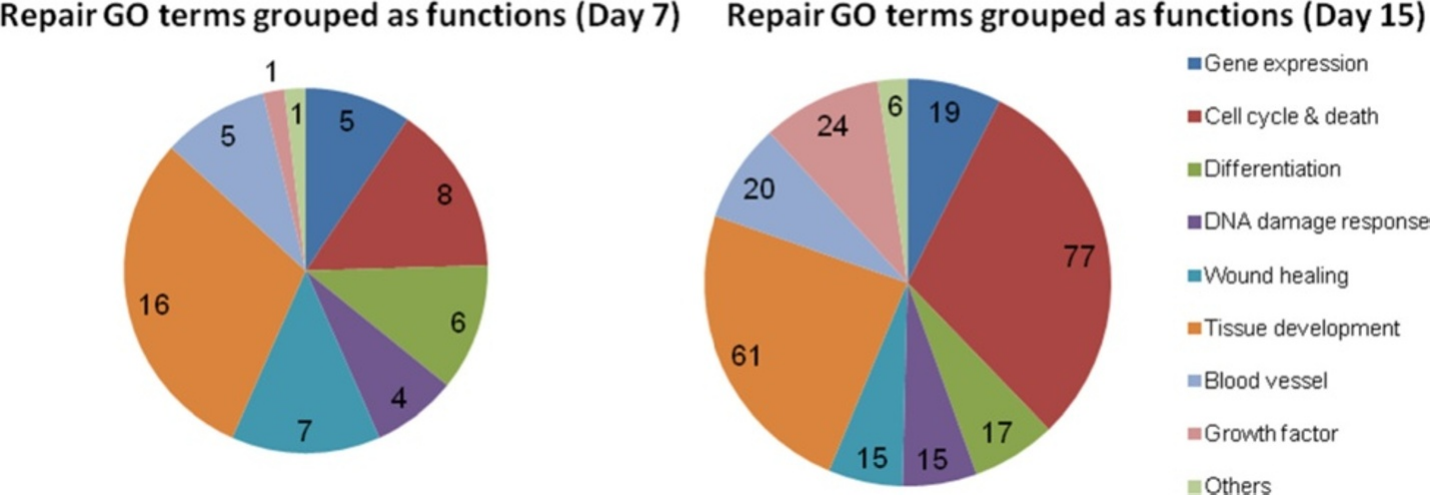
**Summary of lung repair-associated gene functions targeted by their associated miRNAs at 7 and 15 dpi.** Lung repair-related genes based on their summarized functions (GO) showed that more repair functions occurred on day 15. There was a marked increase in the proportion of cell cycle, cell death, and growth factor-related genes, accompanied by a decrease of differentiation and wound healing genes.

The GO term analyses applied after the initial manual selection revealed different processes occurring at each time-point (Figure 4). At 7 dpi, only a relatively small number of 53 GO terms were identified to be directly involved in repair, mainly in tissue development, cell cycle, cell death, and wound healing, implying minimal repair and developmental functions during active viral clearance. In contrast, 254 GO terms were found to be associated directly with repair and development at 15 dpi. Many of them were predominantly involved mainly in cell cycle, cell death, tissue development and growth factors, which corroborates the repair and replacement of damaged lung tissue occurring at 15 dpi.

### TargetScan reveals the close relationship between miRNAs and selected repair-related genes

**Figure 5:**
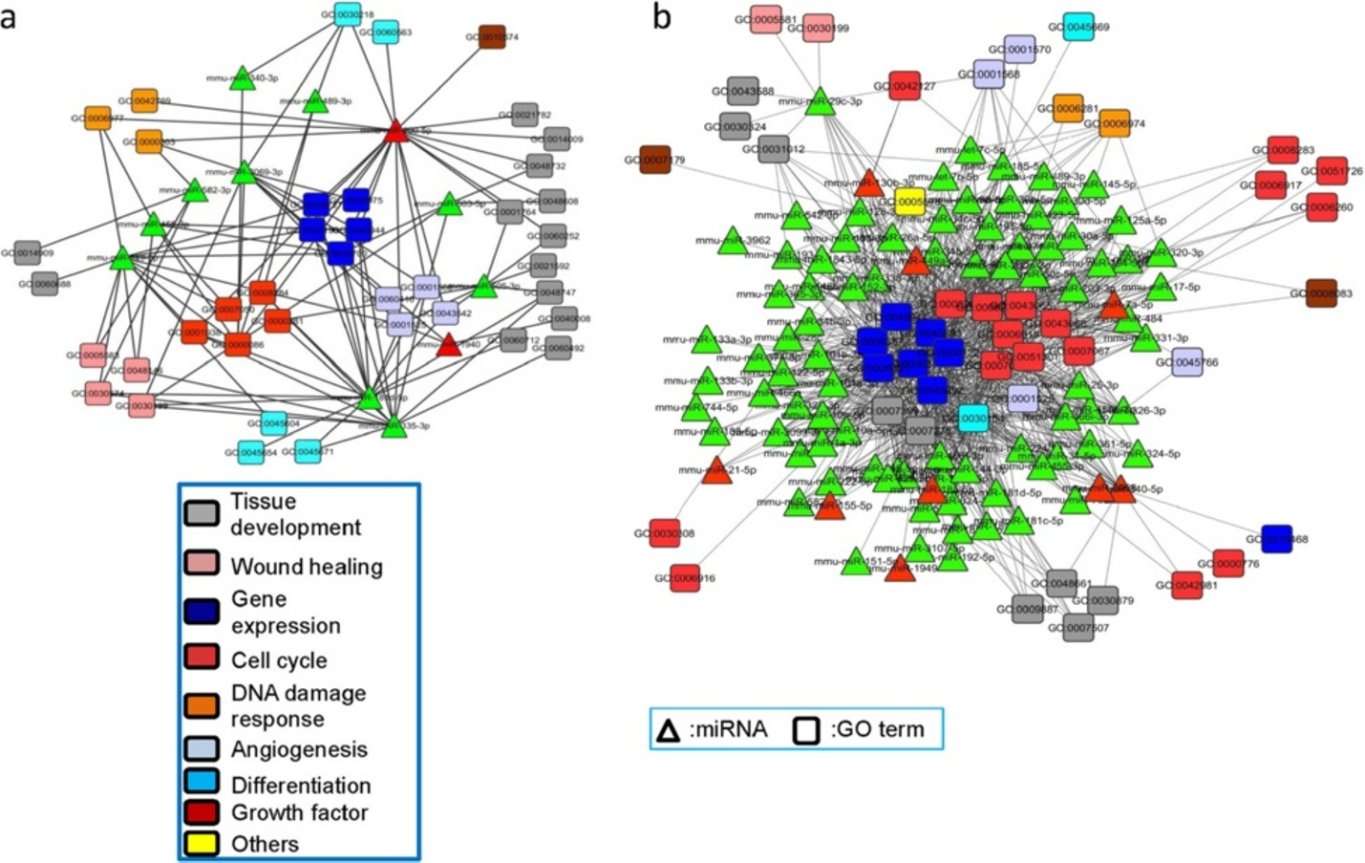
**Further refinement of miRNA selection to identify physiologically important miRNAs during repair.** Integrated miRNA and gene (GO term) networks of selected miRNA candidates. **(a)** At 7 dpi, the network was relatively moderate, with a focus on targeting tissue development. **(b)** At 15 dpi, an extensive network was involved in pulmonary repair and regeneration mainly by targeting cell cycle functions. It is likely that most miRNAs were down-regulated possibly to facilitate developmental gene expression.

To evaluate the extent to which miRNAs were involved with gene regulation in repair and regeneration, TargetScan analyses of the DE miRNAs targeting the selected genes related to repair and regeneration were applied after the GO analyses. Thus, the DE genes were further narrowed down to 61 up- and 43 down-regulated genes at 7 dpi, targeted by the corresponding 2 up- and 10 down-regulated miRNAs. At 15 dpi, there were 556 up- and 193 down-regulated repair-related genes targeted by the corresponding 12 up- and 91 down-regulated miRNAs. When the DE miRNA and repair-associated genes were paired by TargetScan, we thus observed 12 miRNAs at 7 dpi and 103 miRNAs at 15 dpi highly involved in targeting repair-associated genes that were differentially regulated at both time-points. Figure 5 shows the major targets of the altered miRNAs grouped into their respective GO terms. The high frequency of repair GO terms targeting genes in cell cycle, differentiation, wound healing, tissue development and angiogenesis, implied that the repair and regeneration of lung tissues following influenza pneumonia were tightly modulated by miRNAs.

### Selection of candidates having high association with lung repair functions

**Figure 6:**
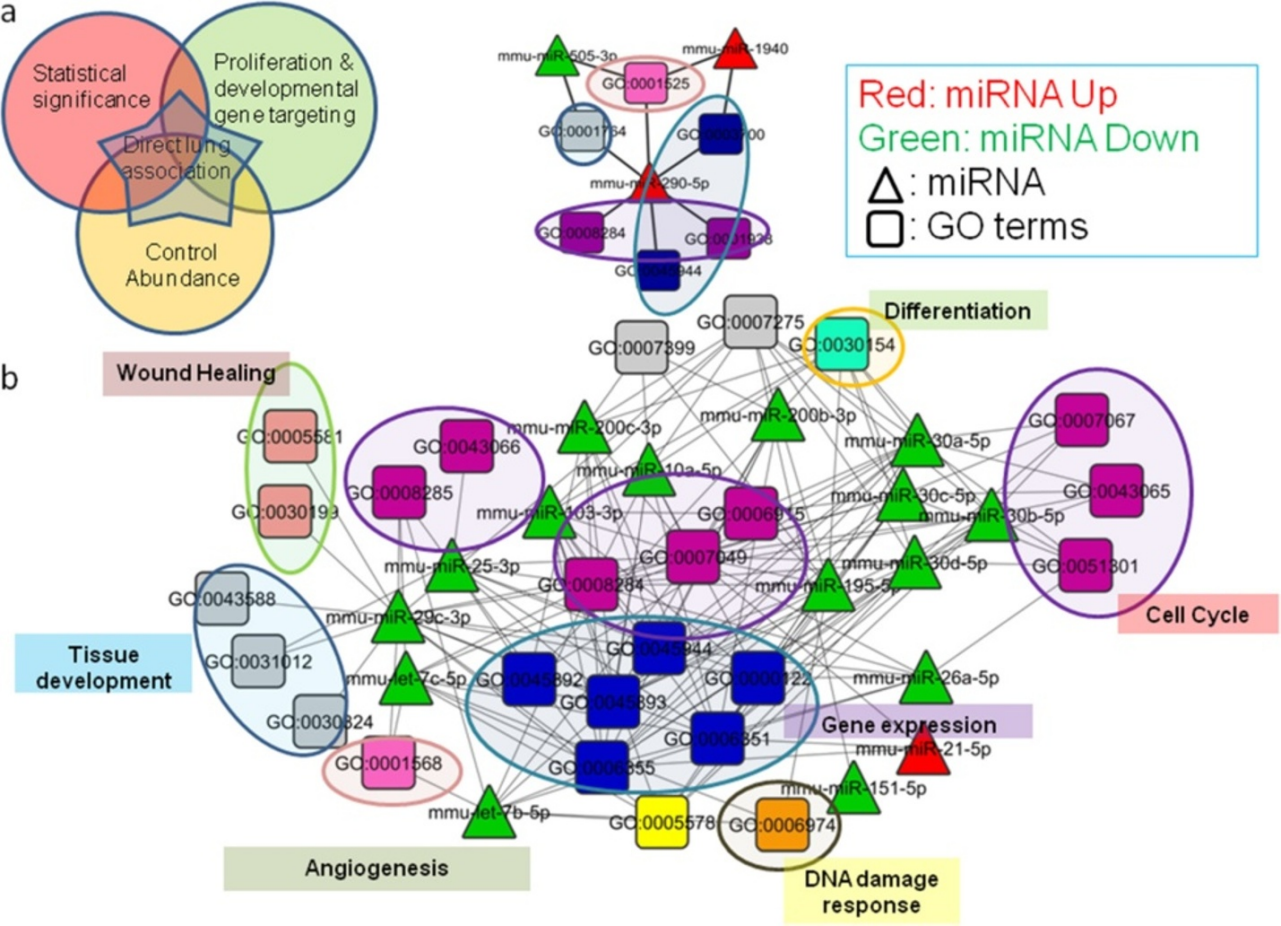
**Targets of the top 20 miRNA candidates in lung repair. (a)** Selection criteria to filter physiologically important miRNAs associated with lung repair for further characterization. **(b)** Most of the GO term targets of the top 20 miRNAs were involved in cell cycle and gene expression. The predominant down-regulation of most of the top 20 miRNAs likely permits enhanced expression of their respective gene targets during pulmonary repair. miR-99a was not included since it targets only a small number of mRNAs.

To screen for the final list of potential candidate miRNA regulators of repair and regeneration, we applied other criteria (Figure 6a). The rationale of this additional filter is that miRNAs changes are usually subtle, and therefore selecting those significantly DE miRNAs with the highest or lowest raw abundance in the control samples would likely culminate in a larger magnitude of change and effect in response to infection. These additional criteria narrowed the selection down to 3 miRNAs at 7 dpi (miR-290, miR-1940, miR-505) which were marked to be crucial in the early repair phase. At 15 dpi, 17 miRNAs (let-7b,c, miR-10a, miR-21, miR-25, miR-26a, miR-29c, miR-30a,b,c,d, miR-99a, miR-103, miR-151, miR-195, and miR-200b,c) were identified to be important in the late repair phase. Most of these miRNAs were also found to target genes in lung developmental GO terms, rendering them the most likely regulators of the transcriptome in influenza-induced pulmonary repair (Figure 6b).

### Possible functions of high-scoring miRNAs involved in targeting the lung repair pathway

Of the 20 shortlisted miRNAs, their putative functions associated with lung repair were inferred through literature searches. (Additional file 2: Table S4) indicates that the DE miRNAs on days 7 and 15 largely differ in their functions. The miRNAs at 7 dpi were engaged in endothelial and fibroblast proliferation (wound healing), whereas miRNAs at 15 dpi were more broadly involved in cell proliferation, apoptosis, tumor suppression and DNA repair, consistent with the functional annotation. The full list of targets of these shortlisted miRNAs is provided in Additional file 3: Table S1.

## Discussion

miRNAs are believed to play crucial roles during the repair of the lungs following injury. We therefore identified miRNAs that are differentially regulated in a time-dependent manner during the early and later recovery phases of the lungs using a murine influenza infection model. The recovery time-points were selected based on previously documented kinetics of lung-repairing cell types initially present on day 7 (early phase) and that peak on day 15 (late phase) [10, 12]. Our *in vivo* investigation unveiled potential candidate miRNAs strongly associated with pulmonary repair and regeneration after acute inflammatory lung injury due to influenza pneumonia.

### Marked expression of repair, proliferation and differentiation genes after viral clearance at 15 dpi

At 7 dpi, we observed highly specific differential expression of mainly inflammatory and viral innate immune response-related genes in the lungs. This likely reflects the specific commitment of damaged lungs to combat viral infection rather than for repair functions. In addition, the respiratory burst of innate immune responses also causes oxidative stress and DNA damage, culminating in greater genetic instability and apoptosis which hamper cellular proliferation for replacing damaged cells [24–26]. However, day 15 corresponds to the later phase of recovery, characterized by viral clearance e.g. by lymphocytes in the lungs to execute more specific adaptive responses. The emergence of new immature epithelial cells as indicated by histologic analyses as well as increased PCNA-positive cells also suggest re-epithelialization of lung tissues following viral clearance. This was evident from the patches of PCNA-positive cells within areas devoid of SP-C-positive cells, which may reflect epithelial progenitor cells or lung stem cells prior to differentiating into new pneumocytes to replace the damaged ones. Regeneration was also illustrated by the significantly increased double-positive cells lining the edges of these SP-C-deprived areas. Therefore, more efficient repair processes would be expected at 15 dpi, with less oxidative stress when the respiratory burst subsided in the lungs. Alveolar epithelial cells could thus be replaced more readily since repair would less likely be compromised by DNA damage and mutation [26].

### Potential regulatory roles of specific miRNAs in lung repair and development

The number of DE miRNAs was directly proportional to the number of DE genes involved in repair and development. Most DE miRNAs directly target repair-associated genes, especially at 15 dpi when extensive repair occurred. The close association between miRNAs and lung development has been documented [13, 19, 27–29]. The differentially regulated miRNAs were unique with almost no overlap between the two time-points, indicating that miRNA expression changed transiently according to the temporal gene expression requirements in the lungs. The top 20 miRNA candidates were identified, together with their roles and targets during the lung repair phases following influenza infection. Some of these miRNAs are highly involved in development, regeneration, and cancer progression due to their apparent roles in proliferation and tumor suppression. This underscores that tissue repair entails essentially tightly controlled proliferation as opposed to uncontrolled proliferation in cancer which involves similar factors [19].

We therefore postulate that the increase of certain stem cell-associated miRNAs (e.g. miR-290) promotes the stemness of cells during influenza-induced lung repair [30–32]. The let-7 miRNAs are implicated in developmental functions and cellular proliferation, and also regulate and inhibit proliferation in a cancer context. Therefore, their transiently diminished expression during repair may facilitate the proliferation of new tissues [33–35]. The miR-200 family is often enriched in epithelial tissues, and their decreased expression is linked to epithelial-mesenchymal transition and cell stemness. Hence, their reduced expression may suggest enhanced plasticity of epithelial cells in repairing lungs to replace damaged areas [36]. Lowered miR-200 expression may also augment endothelial development to repair the lung vasculature [37]. Interestingly, Li et al [38] found that miR-200a is differentially expressed in influenza virus infection, and that miRNA expression profiles vary depending on the virus strains. miR-21 targets proliferation-suppressing factors, and its elevated expression during repair coincides with increased proliferation in repairing lungs [39]. Notably, increased miR-21 may cause detrimental effects due to their targets in the TGF-β pathway, suggesting that miR-21 knockdown may prevent lung complications such as fibrosis [40]. On the other hand, some other miRNAs are possibly involved in the regulation and surveillance of repair to prevent uncontrolled cell proliferation. Thus, members of the miR-30 family were significantly down-regulated so that expression of its main target p53 could be suitably elevated to counteract the higher proliferation in recovering lung tissues, which are more prone to DNA damage and mutation in the presence of increased DNA synthesis [41]. Hence, miR-30 appears to act as a tumor suppressor, with its subdued expression facilitating proliferation, but concurrently activating the negative feedback loop of p53, thus showcasing the intricate roles that miRNAs play in pulmonary damage and repair [42].

The functions of the physiologically important miRNAs during tissue repair may reflect an intermediate state between organismal development and cancer progression. Transient and controlled changes in expression of proliferation activators and inhibitors may lead to a state akin to development in contrast to cancer progression, as highlighted by the vastly different DE miRNA profiles between days 7 and 15.

### miRNAs and their potential applications in lung tissue damage and repair

This study revealed that the regulation of miRNAs was unique during different phases of repair after influenza infection. The regulatory roles of these miRNAs in tissue repair can be tested for their veracity in improving lung repair and the overall outcome of influenza pneumonia. For example, due to the nature of miRNA targeting, some of these miRNAs (such as miR-21 and let-7) may even serve dual roles of limiting damage and accelerating repair as they possess anti-inflammatory properties besides regulating pulmonary repair activities, i.e. they may minimize damage from inflammation secondary to infection [43, 44]. Therefore, future studies are warranted to further characterize the potential miRNA candidates *in vitro* and *in vivo*, in order to determine their detailed roles in ALI and repair following influenza pneumonia.

## Conclusion

We anticipate that many of the biological processes observed during influenza lung damage and repair may also be relevant to other types of viral infection and lung injury, and will therefore contribute to our basic understanding of tissue repair of damaged lungs. Ultimately, this integrative systems biology approach highlights the significant roles of the miRNAs in acute inflammatory lung injury and regeneration, and revealed certain miRNAs that may be instrumental in the repair and reconstruction of the lung architecture post-infection.

## Methods

### Virus strain and infection of animals

All animal experiments with 6–8 week old female BALB/c mice were approved by the Institutional Animal Care and Use Committee (IACUC), National University of Singapore (protocol 050/11), and fulfilled all listed criteria in the ARRIVE guidelines (Additional file 4). Mice were housed in ABSL-2 facilities in ventilated cages according to IACUC guidelines. Animals were divided into 4 groups each consisting of 4 mice, i.e. control uninfected and infected groups at 7 and 15 dpi. The two time-points were chosen based on the kinetics of influenza infection, i.e. when repair of primary viral damage commences and certain repair cell types initially appear at 7 dpi, whereas repair of inflammatory damage begins after viral clearance and when repair cell types peak at 15 dpi [10, 12]. Mice were anesthetized with a mixture of 7.5 mg/ml ketamine and 0.1 mg/ml medetomidine, and infected intra-tracheally with a sub-lethal dose of 23 plaque-forming units of influenza A/Puerto Rico/8/1934(H1N1) or PR8 virus (in 50 μl volume) to ensure that all mice recovered from the infection. Control mice were given phosphate-buffered saline (PBS) intra-tracheally. Mice were monitored daily for weight loss from infection and weight gain from recovery. Upon reaching the stipulated time-point, mice were euthanized in a CO_2_ chamber, and the lungs harvested for histology and RNA extraction.

### Murine lung tissue processing and extraction of miRNAs and mRNAs

Approximately 10% of each lobe of lung was snap-frozen at -80°C to preserve RNAs. The lungs were then homogenized in Trizol solution (Qiagen, Hilden, Germany) using a gentleMACS tissue dissociator (Miltenyi Biotec, Bergisch Gladbach, Germany), and total RNA was immediately extracted using the miRNeasy miRNA extraction kit (Qiagen). The isolated total RNA samples containing miRNAs were then subjected to miRNA and gene microarray analyses.

### Histolopathologic analyses

The remaining 90% of each lung was fixed in 4% formalin, dehydrated in ascending ethanol concentrations, embedded in paraffin, sectioned into 4-μm slices, and stained with hematoxylin and eosin (H&E). Ideally, the lungs could be inflated prior to fixation. Notwithstanding this, highly satisfactory assessment of the infected areas was achieved by an experienced pulmonary pathologist to include: inflammatory cell infiltration (polymorphs and lymphocytes); septae, hyaline and fibrin; hemorrhage; edema (for tissue damage); and new progenitor cells and re-epithelization (for tissue repair and replacement). This evaluation was then correlated with the molecular analyses. Scoring of damage to ascertain the homogeneity of the infection was also conducted for mouse lungs at 7 dpi based on a modified scoring system, i.e.% infected × [alveolar hemorrhage + 2(alveolar inflitrates) + 3(fibrin) + alveolar septal congestion] [23]. The scoring was carried out based on the worst-affected area of the lungs, which then yielded the percentage of the lung area showing similar scores. Since mice at 15 dpi were recovering and could not be scored with the same system, the percentage of lung area affected by the infection was obtained instead to ascertain the homogeneity of damage.

### Immunohistochemistry (IHC)

Paraffin-embedded blocks of lung tissues were also sectioned into 5-μm slices for IHC analyses. The slides were dewaxed with two washes of xylene, rehydrated with two washes of 100% ethanol, and one wash each of 90%, 70% and 50% ethanol, followed by two washes of deionized water. Slides were then subjected to antigen retrieval in boiling sodium citrate buffer with Tween 20. Slides were further permeated with 0.025% of Triton X-100 for 10 min, before being blocked with 3% BSA for 2 h. Primary antibodies for PCNA (SC-9857, Santa Cruz Biotechnology, Dallas, TX) and SP-C (SC-13979, Santa Cruz Biotechnology) at 100× dilution in 3% BSA were used for co-staining dividing and repairing cells and AT2 pneumocytes, respectively. Following overnight incubation at 4°C in the dark, the slides were washed with Tris-buffered saline thrice for 5 min each, and stained with secondary antibody containing Alexa Fluor 488 for PCNA and Alexa Fluor 568 for SP-C (Molecular Probes, Grand Island, NY) at room temperature for 1 h in the dark. Slides were then washed and mounted in colloidal gold Antifade mounting medium with DAPI (Molecular Probes). Slides were then concurrently scanned with the high-resolution MIRAX MIDI system equipped with fluorescence illumination (Carl Zeiss, Jena, Germany) for the complete lung image to quantify dividing AT2 pneumocytes. Stained sections were also visualized with a FX-1000 confocal microscope at higher magnification.

### Image analyses for quantification of dividing AT2 pneumocytes in the lungs

An automated computer algorithm was developed to count the number of total cells, PCNA-positive cells, and PCNA-positive AT2 pneumocytes. Nuclei were segmented by converting the DAPI fluorescence channel into black-and-white using manual thresholding of DAPI intensities, and removing areas less than 3 pixels in size. The number of nuclei was counted as the number of total cells. The PCNA fluorescence channel was next converted to black-and-white using manual thresholding of PCNA intensities. PCNA-positive nuclei were identified by selecting co-localized areas of nuclei and PCNA channel, and performing dilating-hole filling-eroding using “disk” operator with 3 pixels in size. The number of PCNA-positive nuclei was counted as the number of PCNA-positive cells. Finally, the SP-C fluorescence channel was converted into black-and-white using manual thresholding of SP-C intensities, and areas less than 2 pixels in size were ignored. The surrounding areas of PCNA-positive nuclei (1 pixel in radius) were then checked for SP-C signal in the SP-C channel. The number of PCNA-positive nuclei with surrounding SP-C signal was counted as the number of PCNA-positive AT2 cells. All image processing and computation algorithms were implemented using MATLAB with image processing toolbox (MathWorks, Natick, MA). The MATLAB codes are available upon request.

### miRNA and gene microarray analyses

Extracted total RNAs containing miRNAs were subjected to quality control using a bio-analyzer to ensure proper RNA quality before the microarray experiments. Total RNA samples from 4 mice per group were then hybridized individually onto the respective microarray platforms (Agilent, Santa Clara, CA). The miRNA microarray was a Mouse miRNA, 8 × 60 K format platform (AMADID 38112), while the gene microarray was a SurePrint G3 Mouse GE, 8 × 60 K, 1 color format platform (AMADID 028005). A total of 32 arrays (i.e. 16 miRNA arrays and 16 mRNA arrays) were hybridized to total RNA samples extracted from the lungs of 4 infected mice and 4 control mice for each 7 and 15 dpi. The expression values of the array probes were calculated following the manufacturer’s standard array protocol. Only probes above the expression threshold of 20 (raw signal intensity) were used to derive expression values.

The expression data for the filtered probes of both miRNA (1,179 probes) and gene (55,681 probes) microarrays were normalized using quantile normalization, and were subjected to DE analyses using QPROT software (an extension of the QSPEC software for generic expression data with missing values). DE genes were identified using the Z-statistics threshold that controls the FDR at 5% [45]. The DE miRNAs and genes were further analyzed below to shortlist candidates associated with active roles in pulmonary repair and regeneration.

### Real-time RT-PCR validation of miRNA and gene expression

A total of 5 miRNAs and 20 genes that were significantly regulated at both 7 and 15 dpi were selected for real-time RT-PCR to validate their differential expression based on the microarray data. A stem-loop primer miRNA Universal TaqMan RT-PCR system (Applied Biosystems, Foster City, CA) was utilized to validate miRNAs, and normalized using murine SNORD68 small nucleolar RNA [46, 47]. The miRNA stem-loop primers, specific forward primers and universal reverse primer are listed in Additional file 5: Table S2. Real-time RT-PCR assays to validate 20 genes were performed using reverse transcription with random hexamers (Promega, Madison, WI), the SYBR Green Master mix and LightCycler real-time PCR system (Roche, Penzberg, Germany), normalized against the RPL13a gene (Additional file 6: Table S3).

### Developmental pathway and TargetScan analyses

The DE genes identified above were filtered for repair-associated genes by the following criteria. Functional selection for repair association was performed manually using the function tree of the IPA software (Qiagen), where genes under developmental and repair functional groups were identified. These genes were then filtered again with the hypergeometric test and FDR control (the Benjamini-Hochberg procedure), and GO analysis [48]. A concurrent miRNA TargetScan analysis at a PCT score of 0.5 was performed using the GeneSpring GX software (Agilent), and only miRNAs that target the filtered genes from the previous steps were selected. The selected genes (grouped as GO terms at P < 0.05) and miRNAs were then visualized in a network with Cytoscape software (v3.0.1).

### Further refinement of miRNA candidate selection

To further narrow down the selected miRNA candidates, an additional filter system of miRNA raw abundance in control samples was taken into account. Therefore, a final set of miRNAs was selected based on their statistical significance, targeting related GO term members of gene analysis, highest or lowest raw abundance in control samples where the fold changes were deemed more significant. The selected miRNAs were then classified based on their expression changes and their implicated functions, and also whether they target genes directly associated with lung development and regeneration.

### Statistical analyses

FDR calculation and GO enrichment analyses of the array dataset were performed using R statistics software for selection of significant DE miRNAs and genes. Student’s one sample t-test was performed on real-time RT-PCR verification and dividing AT2 pneumocyte quantification data with GraphPad and SPSS software. Results were represented as mean ± standard deviation (SD).

## Electronic supplementary material

Additional file 1: Figure S1: Scatter plot showing between-sample correlation of probe-set intensity in infected groups (miRNA and mRNA expression at both 7 and 15 dpi). (TIFF 209 KB)

Additional file 2: Table S4: miRNAs of higher significance based on selection criteria. Implicated functions of 20 shortlisted DE miRNAs. (DOCX 45 KB)

Additional file 3: Table S1: Significantly DE mRNAs targeted by the top 20 shortlisted miRNAs (with directional changes). (XLSX 28 KB)

Additional file 4: **ARRIVE guideline checklist.** (PDF 1 MB)

Additional file 5: Table S2: Sequences (5′-3′) of stem-loop RT primers, real-time specific forward primers and real-time universal reverse primer for miRNA RT-PCR validation. (DOCX 12 KB)

Additional file 6: Table S3: Sequences (5′–3′) of forward and reverse primers for real-time quantitative RT-PCR of selected genes. (DOCX 13 KB)

## Abbreviations

ALI: Acute lung injury
miRNA: MicroRNA
dpi: Days post-infection
PCNA: Proliferating cell nuclear antigen
SP-C: Surfactant protein-C
AT2: Alveolar type 2
FDR: False discovery rate
DE: Differentially expressed
IPA: Ingenuity pathway analysis
GO: Gene ontology
IACUC: Institutional animal care and use committee
PBS: Phosphate-buffered saline
H&E: Hematoxylin and eosin
IHC: Immunohistochemistry
SD: Standard deviation.
