# Supplementary figures and images for: Micro-RNAs in regenerating lungs: an integrative systems biology analysis of murine influenza pneumonia
Source: BMC Genomics. 2014 Jul 11;15(1):587. doi: 10.1186/1471-2164-15-587 (PMC4108790; doi:10.1186/1471-2164-15-587)

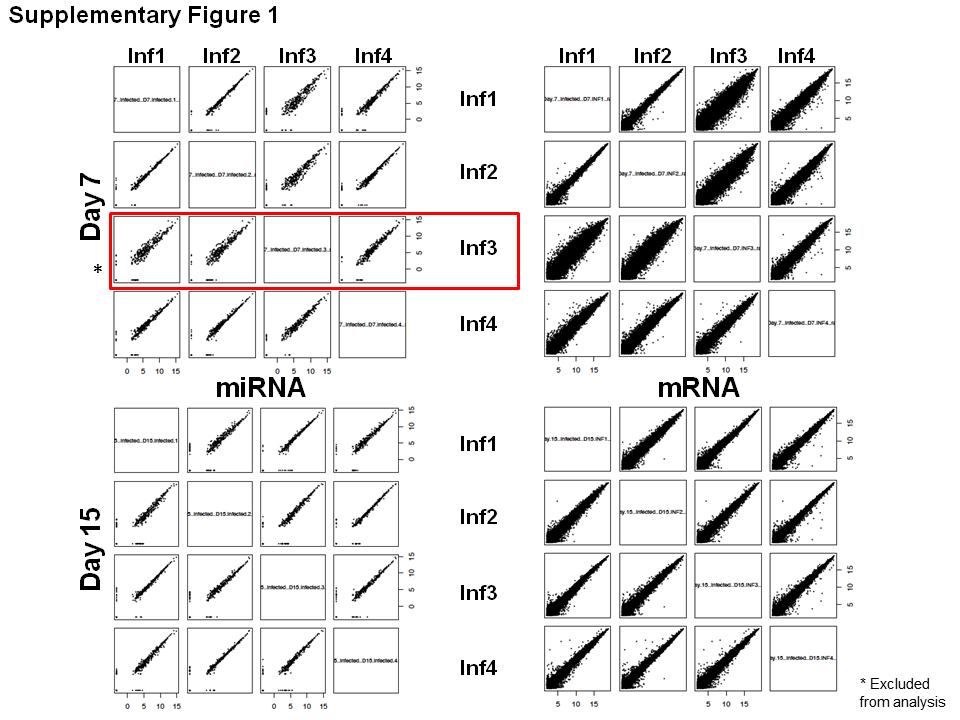

Supplement: Supplementary file 1 — Additional file 1: Figure S1: Scatter plot showing between-sample correlation of probe-set intensity in infected groups (miRNA and mRNA expression at both 7 and 15 dpi). (TIFF 209 KB) [file 12864_2013_6268_MOESM1_ESM.tiff]
