# Supplementary material for: Micro-RNAs in regenerating lungs: an integrative systems biology analysis of murine influenza pneumonia
Source: BMC Genomics. 2014 Jul 11;15(1):587. doi: 10.1186/1471-2164-15-587 (PMC4108790; doi:10.1186/1471-2164-15-587)
Supplement: Supplementary file 2 — Additional file 2: Table S4: miRNAs of higher significance based on selection criteria. Implicated functions of 20 shortlisted DE miRNAs. (DOCX 45 KB) [file 12864_2013_6268_MOESM2_ESM.docx]

**Table S4. miRNAs of higher significance based on selection criteria**

| ***miRNAs at 7 dpi*** | ***Sig. target***  ***gene count*** | ***Validated target***  ***sig. gene*** | ***Experimentally validated functions [references]*** |
| --- | --- | --- | --- |
| ↑ mmu-miR-290-5p | 21 |  | Stem cells generation, endothelial [[1](#_ENREF_1)] |
| ↑ mmu-miR-1940 | 12 |  | Flu related [[2](#_ENREF_2)] |
| ↓ mmu-miR-505-3p | 9 |  | Undefined |

| ***miRNAs at 15 dpi*** |  |  |  |
| --- | --- | --- | --- |
| ↑ mmu-miR-21-5p * | 43 | RECK [[3](#_ENREF_3)] | Proliferation factor [[4](#_ENREF_4)] |
| ↓ mmu-let-7b-5p * | 66 | Mycn [[6](#_ENREF_6)] | Tumor suppressor [[5](#_ENREF_5)] |
| ↓ mmu-let-7c-5p * | 66 |  |  |
| ↓ mmu-miR-10a-5p * | 57 |  | Stem cell differentiation factor [[7](#_ENREF_7)] |
| ↓ mmu-miR-25-3p * | 59 |  | Tumor suppressor [[8](#_ENREF_8), [9](#_ENREF_9)] |
| ↓ mmu-miR-26a-5p * | 74 | Ezh2 [[10](#_ENREF_10)] | Tumor suppressor [[11](#_ENREF_11)] ; Angiogenesis suppressor [[12](#_ENREF_12)] |
| ↓ mmu-miR-29c-3p * | 70 | Cav2 [[13](#_ENREF_13)] | Tumor suppresor [[14](#_ENREF_14)]; Apoptosis factor |
| ↓ mmu-miR-30a-5p * | 76 |  | Tumor suppressor [[15](#_ENREF_15)]; Cell surveillance [[16](#_ENREF_16)] |
| ↓ mmu-miR-30b-5p * | 80 |  |  |
| ↓ mmu-miR-30c-5p * | 81 |  |  |
| ↓ mmu-miR-30d-5p * | 75 |  |  |
| ↓ mmu-miR-99a-5p * | 10 |  | Tumor suppressor [[17](#_ENREF_17)]; Keratinocyte/epithelium proliferation suppressor [[18](#_ENREF_18)] |
| ↓ mmu-miR-103-3p * | 88 |  | Cell migratory factor [[19](#_ENREF_19), [20](#_ENREF_20)] |
| ↓ mmu-miR-151-5p * | 20 |  | Undefined |
| ↓ mmu-miR-195-5p * | 96 | E2f3 [[21](#_ENREF_21)] | Tumor suppressor [[22](#_ENREF_22)]; Stem cell Apoptotic factor [[23](#_ENREF_23)] |
| ↓ mmu-miR-200b-3p * | 80 |  | Epithelial and endothelial maintenance [[24](#_ENREF_24), [25](#_ENREF_25)] |
| ↓ mmu-miR-200c-3p * | 80 |  |  |

Note: The 20 top DE miRNAs and their respective significantly DE target mRNAs. The associated miRNA functions were obtained via literature review on experimentally observed miRNA functions in different systems. Most miRNAs in day 7 showed no direct association with lung repair functions, whereas DE miRNAs at day 15 exhibited greater association with lung repair and proliferation. Asterisks denote direct lung repair association.

References:

1. Kaspi H, Chapnik E, Levy M *et al.* miR--290--295 Regulate Embryonic Stem Cell Differentiation Propensities by Repressing Pax6. *Stem Cells* 2013.

2. Zhang Z, Hu S, Li Z *et al.* Multiple amino acid substitutions involved in enhanced pathogenicity of LPAI H9N2 in mice. *Infect Genet Evol* 2011; **11**:1790-1797.

3. Hu SJ, Ren G, Liu JL *et al.* MicroRNA expression and regulation in mouse uterus during embryo implantation. *J Biol Chem* 2008; **283**:23473-23484.

4. Zhong Z, Dong Z, Yang LGong Z. miR-21 induces cell cycle at S phase and modulates cell proliferation by down-regulating hMSH2 in lung cancer. *J Cancer Res Clin Oncol* 2012; **138**:1781-1788.

5. Cimadamore F, Amador-Arjona A, Chen C, Huang CTTerskikh AV. SOX2-LIN28/let-7 pathway regulates proliferation and neurogenesis in neural precursors. *Proc Natl Acad Sci U S A* 2013; **110**:E3017-3026.

6. Melton C, Judson RLBlelloch R. Opposing microRNA families regulate self-renewal in mouse embryonic stem cells. *Nature* 2010; **463**:621-626.

7. Li J, Dong J, Zhang ZH *et al.* miR-10a restores human mesenchymal stem cell differentiation by repressing KLF4. *J Cell Physiol* 2013; **228**:2324-2336.

8. Li Q, Zou C, Han Z *et al.* MicroRNA-25 functions as a potential tumor suppressor in colon cancer by targeting Smad7. *Cancer Lett* 2013; **335**:168-174.

9. Xu JY, Yang LL, Ma C *et al.* MiR-25-3p attenuates the proliferation of tongue squamous cell carcinoma cell line Tca8113. *Asian Pac J Trop Med* 2013; **6**:743-747.

10. Wong CFTellam RL. MicroRNA-26a targets the histone methyltransferase Enhancer of Zeste homolog 2 during myogenesis. *J Biol Chem* 2008; **283**:9836-9843.

11. Fu X, Meng Z, Liang W *et al.* miR-26a enhances miRNA biogenesis by targeting Lin28B and Zcchc11 to suppress tumor growth and metastasis. *Oncogene* 2013.

12. Icli B, Wara AK, Moslehi J *et al.* MicroRNA-26a Regulates Pathological And Physiological Angiogenesis by Targeting BMP/SMAD1 Signaling. *Circ Res* 2013.

13. He A, Zhu L, Gupta N, Chang YFang F. Overexpression of micro ribonucleic acid 29, highly up-regulated in diabetic rats, leads to insulin resistance in 3T3-L1 adipocytes. *Mol Endocrinol* 2007; **21**:2785-2794.

14. Wang H, Zhu Y, Zhao M *et al.* miRNA-29c Suppresses Lung Cancer Cell Adhesion to Extracellular Matrix and Metastasis by Targeting Integrin beta1 and Matrix Metalloproteinase2 (MMP2). *PLoS One* 2013; **8**:e70192.

15. Kao CJ, Martiniez A, Shi XB *et al.* miR-30 as a tumor suppressor connects EGF/Src signal to ERG and EMT. *Oncogene* 2013.

16. Li J, Donath S, Li Y *et al.* miR-30 regulates mitochondrial fission through targeting p53 and the dynamin-related protein-1 pathway. *PLoS genetics* 2010; **6**:e1000795.

17. Kuo YZ, Tai YH, Lo HI *et al.* MiR-99a exerts anti-metastasis through inhibiting myotubularin-related protein 3 expression in oral cancer. *Oral Dis* 2013.

18. Jin Y, Tymen SD, Chen D *et al.* MicroRNA-99 family targets AKT/mTOR signaling pathway in dermal wound healing. *PLoS One* 2013; **8**:e64434.

19. Chen HY, Lin YM, Chung HC *et al.* miR-103/107 promote metastasis of colorectal cancer by targeting the metastasis suppressors DAPK and KLF4. *Cancer research* 2012; **72**:3631-3641.

20. Moncini S, Salvi A, Zuccotti P *et al.* The role of miR-103 and miR-107 in regulation of CDK5R1 expression and in cellular migration. *PLoS One* 2011; **6**:e20038.

21. Xu T, Zhu Y, Xiong Y *et al.* MicroRNA-195 suppresses tumorigenicity and regulates G1/S transition of human hepatocellular carcinoma cells. *Hepatology* 2009; **50**:113-121.

22. Fu MG, Li S, Yu TT *et al.* Differential expression of miR-195 in esophageal squamous cell carcinoma and miR-195 expression inhibits tumor cell proliferation and invasion by targeting of Cdc42. *FEBS Lett* 2013.

23. Zhou Y, Jiang H, Gu J *et al.* MicroRNA-195 targets ADP-ribosylation factor-like protein 2 to induce apoptosis in human embryonic stem cell-derived neural progenitor cells. *Cell Death Dis* 2013; **4**:e695.

24. Chan YC, Roy S, Khanna SSen CK. Downregulation of endothelial microRNA-200b supports cutaneous wound angiogenesis by desilencing GATA binding protein 2 and vascular endothelial growth factor receptor 2. *Arterioscler Thromb Vasc Biol* 2012; **32**:1372-1382.

25. Peng C, Li N, Ng YK *et al.* A unilateral negative feedback loop between miR-200 microRNAs and Sox2/E2F3 controls neural progenitor cell-cycle exit and differentiation. *J Neurosci* 2012; **32**:13292-13308.
