# Supplementary material for: Micro-RNAs in regenerating lungs: an integrative systems biology analysis of murine influenza pneumonia
Source: BMC Genomics. 2014 Jul 11;15(1):587. doi: 10.1186/1471-2164-15-587 (PMC4108790; doi:10.1186/1471-2164-15-587)
Supplement: Supplementary file 5 — Additional file 5: Table S2: Sequences (5′-3′) of stem-loop RT primers, real-time specific forward primers and real-time universal reverse primer for miRNA RT-PCR validation. (DOCX 12 KB) [file 12864_2013_6268_MOESM5_ESM.docx]

| *Mouse miRNA* | *RT primer* | *Specific forward primer* | *Universal reverse primer* |
| --- | --- | --- | --- |
| miR-335-3p | GTCGTATCCAGTGCAGGGTCCGAGGTATTCGCAC  TGGATACGACGGTCAG | GCGCGTTTTTCATTATTGCTC | 5′-GTGCAGGGT  CCGAGGT-3′ |
| miR-582-3p | GTCGTATCCAGTGCAGGGTCCGAGGTATTCGCAC  TGGATACGACGTTCAG | GGCCGCTAACCTGTTGAACAA |  |
| miR-34b-3p | GTCGTATCCAGTGCAGGGTCCGAGGTATTCGCAC  TGGATACGACGATGGC | GCGCAATCACTAACTCCAC |  |
| miR-542-5p | GTCGTATCCAGTGCAGGGTCCGAGGTATTCGCAC  TGGATACGACTCGTGA | GCGCCTCGGGGATCATCA |  |
| miR-21-5p | GTCGTATCCAGTGCAGGGTCCGAGGTATTCGCAC  TGGATACGACTCAACA | GCGCTAGCTTATCAGACTG |  |
| SNORD68 (snoRNA202) | GTCGTATCCAGTGCAGGGTCCGAGGTATTCGCAC  TGGATACGACCATCAG | GCTGTACTGACTTGATGAAAG |  |

**Table S2: Sequences (5′-3′) of stem-loop RT primers, real-time specific forward primers and real-time universal reverse primer for miRNA RT-PCR validation**
