# Supplementary material for: Micro-RNAs in regenerating lungs: an integrative systems biology analysis of murine influenza pneumonia
Source: BMC Genomics. 2014 Jul 11;15(1):587. doi: 10.1186/1471-2164-15-587 (PMC4108790; doi:10.1186/1471-2164-15-587)
Supplement: Supplementary file 6 — Additional file 6: Table S3: Sequences (5′–3′) of forward and reverse primers for real-time quantitative RT-PCR of selected genes. (DOCX 13 KB) [file 12864_2013_6268_MOESM6_ESM.docx]

**Table S3: Sequences (5′–3′) of forward and reverse primers for real-time quantitative RT-PCR of selected genes**

| *Gene* | *Forward primer* | *Reverse primer* |
| --- | --- | --- |
| ANGPTL4 | CATCCTGGGACGAGATGAACT | TGACAAGCGTTACCACAGGC |
| PLAT | TGACCAGGGAATACATGGGAG | CTGAGTGGCATTGTACCAGGC |
| GADD45G | GGGAAAGCACTGCACGAACT | AGCACGCAAAAGGTCACATTG |
| GFIIB | ATGCCACGGTCCTTTCTAGTG | GGAAGGCTCTGGTTCAGCAA |
| MMP8 | TCTTCCTCCACACACAGCTTG | CTGCAACCATCGTGGCATTC |
| APLNR | GGTTACAACTACTATGGGGCTGA | AGCTGAGCGTCTCTTTTCGC |
| FAT4 | CAGTGGTGATCCAGGTACGG | TCATGCGCTGTCACGGAAATA |
| CTHRC1 | CAGTTGTCCGCACCGATCA | GGTCCTTGTAGACACATTCCATT |
| SOX4 | GACAGCGACAAGATTCCGTTC | GTTGCCCGACTTCACCTTC |
| ESM1 | CTGGAGCGCCAAATATGCG | TGAGACTGTACGGTAGCAGGT |
| EREG | CTGCCTCTTGGGTCTTGACG | GCGGTACAGTTATCCTCGGATTC |
| GDF6 | TATCGCGCCCCTAGAGTACG | ATGCTAATGGGAGTCAGTTTGG |
| FGF1 | CCCTGACCGAGAGGTTCAAC | GTCCCTTGTCCCATCCACG |
| FST | TGCTGCTACTCTGCCAGTTC | GTGCTGCAACACTCTTCCTTG |
| CDC25C | ATGTCTACAGGACCTATCCCAC | ACCTAAAACTGGGTGCTGAAAC |
| RAD51 | AAGTTTTGGTCCACAGCCTATTT | CGGTGCATAAGCAACAGCC |
| IGFBP6 | GCTGCTAATGCTGTTGTTCGC | GCACTTAGGGCTGTAGACCC |
| NTN4 | GCAGGCTTGAATGGAGTAGC | GCAGCGTTGCATTTATCACAC |
| TGFBR3 | GGTGTGAACTGTCACCGATCA | GTTTAGGATGTGAACCTCCCTTG |
| RPL13a | TACCAGAAAGTTTGCTTACCTGGG | TGCCTGTTTCCGTAACCTCAAG |
| Influenza NS1 | CAGCACTCTTGGTCTGGACA | GGAAGAGAAGGCAATGGTGA |
